# HIV prevalence among transgender women in Northeast Brazil – Findings from two Respondent Driven Sampling studies

**DOI:** 10.1186/s12889-022-14589-5

**Published:** 2022-11-18

**Authors:** Beo Oliveira Leite, Laio Magno, Fabiane Soares, Sarah MacCarthy, Sandra Brignol, Francisco Inácio Bastos, Inês Dourado

**Affiliations:** 1 Collective Health Institute, Federal University of Bahia, Av. Basílio da Gama, s/n, Campus Universitário do Canela, Salvador, Bahia 40110-040 Brazil; 2 Life Sciences Department, Bahia State University, Campus1, Salvador, Bahia Brazil; 3 Department of Health Behavior, Univeristy of Alabama, Birmingham, 1665 University Boulevard, Birmingham, AL 35209 USA; 4 Fluminense Federal University, Rio de Janeiro, RJ Brazil; 5 Oswaldo Cruz Foundation, Rio de Janeiro, RJ Brazil

**Keywords:** HIV, Transgender Women, RDS, Brazil

## Abstract

**Background:**

The HIV epidemic still high among key-populations in Brazil, especially among transgender women (TGW). The aim of this study was to investigate the prevalence of HIV infection among TGW and to analyze factors associated with HIV seropositivity across two cross-sectional surveys conducted in Salvador, Bahia, one of the largest urban centers of Brazil.

**Methods:**

The studies were conducted between 2014 and 2016 and 2016-2017 and employed Respondent-Driven Sampling (RDS) sampling, comprising 127 and 161 TGW residents of Salvador, Bahia. The outcome was the positive rapid antigen testing for HIV infection. Odds ratios (OR) and 95% confidence intervals (95%CI) were obtained using binomial logistic regression.

**Results:**

The HIV prevalence was 9.0% (95%CI: 4.2-18.2) and 24.3% (95%CI: 16.2-34.9). In the first study, factors associated with HIV prevalence were experiencing discrimination by the family (OR 8.22; 95%CI: 1.49-45.48) and by neighbors (OR 6.55; 95%CI: 1.12-38.14) as well as having syphilis (OR 6.56; 95%CI:1.11-38.65); in the subsequent study gender-based discrimination (OR 8.65; 95%CI:1.45-51.59) and having syphilis (OR 3.13; 95%CI: 1.45-51.59) were associated with testing positive for HIV.

**Conclusion:**

We found disproportionately high HIV prevalence among TGW, which underscores the context of vulnerability for this population. The data point to the urgency for intensification and expansion of access to HIV prevention and strategies to stop discrimination in health care and services for this population.

**Supplementary Information:**

The online version contains supplementary material available at 10.1186/s12889-022-14589-5.

## Background

The health of transgender women (TGW) has been increasingly studied, given the urgent health needs of this population [1]. Recent data highlight the unique health needs of TGW, in addition to disproportionately higher rates of HIV infection [2–4] and syphilis [5]. A global systematic review reported an overall prevalence of HIV infection of 19.1% (95%CI:17.4–20.7) among TGW and estimates were 48.78 (95%CI:31.9-76.3) times higher compared to the prevalence among adults in reproductive age in the general population. The estimated prevalence from a set of Brazilian studies conducted between 2000 and 2012 was even higher – 33.1% (95%CI:26.7-39.4) [3]. However, there remains few studies that estimate the more recent prevalence of HIV among TGW in Brazil. In general, existing studies suggest the prevalence is high but varies according to the region and the methodology used by the studies. Across previous studies the prevalence varied from 12 to 32% between 2013 and 2017 [6–9], significantly higher than the general Brazilian female population in 2015 (0.4%) [10].

Such inequities regarding HIV can be explained by contexts of vulnerability that contribute to increased risk for HIV: structural venerability includes poor socioeconomic conditions (e.g., when gender identity represents a barrier to ensuring stable employment) and difficult access to prevention and care services of HIV and other STIs (e.g. discrimination in health services by health professionals and lack of qualified care) [11–15]; interpersonal vulnerability, such as discrimination and violence driven by gender identity (e.g., transphobia) within social interactions creates further vulnerability to HIV [11, 16–19]; Finally, individual vulnerability includes sexual behaviors and practices (e.g., more money offered for higher risk sexual acts such as unprotected sex, condomless anal sex, number of partners or illicit substances use) [3, 11, 18, 20–23].

These data build out the base of evidence for HIV infection among TGW and indicate the urgency of investigations on the determinants of vulnerability to HIV/AIDS. Furthermore, it might points out to the pressing need to increase the availability of culturally-sensitive health services in order to reduce deeply-entrenched stigma and social discrimination, to improve the access to HIV health prevention and treatment services, as well as other health protection and promotion strategies.

[3]. Thus, the aim of this study is to estimate the prevalence of HIV infection among TGW, and to analyze the factors associated with HIV prevalence in two surveys carried out in Salvador, Northeast Brazil.

## Methods

The study analyzes data from two cross-sectional survey with TGW, conducted between September 2014 and April 2016 (PopTrans study) [24, 25], and October 2016 and July 2017 (DIVAS study) [26], respectively. Both studies were carried out in Salvador, the capital of the state of Bahia, which is the fourth largest city in Brazil, the 12th position in the gross domestic product ranking of Brazilian municipalities and has an index of income distribution across a population of 49.0% (46.0-52.0%). The population is mostly composed of Afro-descendant Brazilians (83.1%) [27, 28].

### Study population

The study population, “transgender women”, is commonly defined in the United States and other Western countries as individuals who have a sex assigned at birth that differs from their gender identification or expression [29, 30]. In Brazil, and in some Latin American countries, the terms “*travestis*” or “trans women” are commonly used by individuals and communities themselves too [31–33]. The individuals included in this study are inclusive of all of all diverse gender identities that differ from sex assigned at birth.

Specific study inclusion criteria were: age 15 years or older and 18 years or older respectively in the PopTrans and DIVAS studies; self-identifying as a *travesti*, trans woman or other female gender identity, having been registered as male at birth, and having had at least one sexual intercourse in the last 12 months. The exclusion criteria in both studies were: being under the influence of alcohol or other drugs in the moment people were invited to be interviewed.

### Data collection and sampling

The cross-sectional surveys used Respondent-Driven Sampling (RDS) to recruit study participants. RDS is a chain-link sampling method that begins with “seeds” − a convenience sample of members of the target population chosen by the researchers [34]. Previous qualitative formative research was conducted in both studies to know the population, social meeting places, and the participants who would launch the recruitment process. Focus groups and in-depth interviews were carried out with TGW and initial participants were selected as seeds. Additional formative research was conducted to better assess the heterogeneity of the TGW population, according to demographic and socioeconomic conditions.

In both studies, 5-10 seeds launched the recruitment process. Each seed and subsequent participants received three coupons to invite another TGW from their social contact network (referral chains). For a successful recruitment, RDS includes cash incentives as a compensation for transportation and lost worktime [34].

Data were collected through interviews with a standardized pre-tested questionnaire, conducted face-to-face by interviewers in a space reserved exclusively for this purpose. In both studies, rapid antigen tests for HIV and syphilis were used according to the Brazilian Ministry of Health standards of care for HIV.

### Study variables

The main outcome considered for both studies was HIV infection. All participants received pre- and post-test counseling and guidance before and after receiving the results of their tests. A whole blood rapid test by finger prick was performed. If this rapid test was reactive, a second confirmatory one of a different brand was performed, following the HIV testing algorithm of the Brazilian Ministry of Health [35]. Cases in which the first HIV rapid test was reactive and the second non-reactive or indeterminate, or the first was indeterminate, were considered as inconclusive results and excluded from the analysis [36]. In addition, whole blood rapid test was performed by finger prick for syphilis. Individuals with the first non-reactive test were classified in this study as uninfected. Individuals with both screening and confirmatory tests positive were classified as infected. All participants considered to have a positive rapid test result were referred to public health services for confirmatory tests, monitoring and treatment of the infection. For both studies the HIV and syphilis detection methodologies and procedures were similar and therefore comparable.

The potential predictors of HIV were chosen based on our collective expertise in the field of sexual and gender minority health, and was further complimented by a comprehensive review of the literature: i) sociodemographic: age (< 25 years vs. ≥ 25 years), education (≤ 9 years of study vs. > 9 years), non-white (categorized by the combination of black, brown, yellow and indigenous) and white skin color, income (≤ $190.50 [Brazilian minimum wage per month] vs. > $190.50) and occupation (categorized as (in) formal employment vs. prostitution/unemployment); ii) discrimination: gender-based discrimination (no vs. yes), discrimination in health services (no vs. yes), discrimination in the family (no vs. yes), or discrimination by neighbors (no vs. yes); iii) sexual behavior: condom use during receptive anal sex in the last 6 months (PopTrans) (always use vs. inconsistent use) or condom use during receptive anal sex in the last 30 days (DIVAS) (always use vs. inconsistent use) and having been forced to have sex at some point in their life (no vs. yes); iv) syphilis: history of syphilis infection (no vs. yes).

### Data analysis

Data analysis took into consideration the complex sampling design of the recruitment by RDS methodology, i.e. the dependence between observations resulting from referral chains, and the probabilities of unequal selections due to the different sizes of each participant’s network [37]. For both studies, the seeds were included in the analyses and the unadjusted and adjusted frequencies were estimated for each of the variables. The adjusted was performed according to the RDS-II estimator, proportional to the inverse of the size of each participant’s social network [38]. The magnitude of the associations between the study variables and HIV infection was accessed by the adjusted odds ratios (aOR) with 95% confidence intervals (CI) in the multivariate analyses. The variables included on final model were evaluated for multicollinearity (Supplementary material 1 and 2). Missing, ignored or unanswered information was excluded from the analysis.

The variables with *p*-value ≤0.10 in the bivariate analysis were selected to start modeling and only those with p-value < 0.05 remained in the final model, using backwards procedure. Hosmer-Lemeshow test was used to assess the fit of the final model [39]. The analysis was conducted considering the complex sample design from RDS chain-referral process with linkage using STATA software version 16.0 [40].

## Results

A total of 127 TGW from the PopTrans study and 166 TGW from the DIVAS study were recruited. The HIV prevalence in the PopTrans and DIVAS study was, respectively, 9.0% (95%CI:4.2-18.2) and 24.3% (95%CI:16.2-34.9); and the prevalence of syphilis was, respectively, 31.6% (95%CI 20.2-45.8) and 34.3% (95%CI:26.1-44.9) (Table 1).

**Table 1:** Description of the study population and prevalence of HIV infection in Salvador, Northeast Brazil

Variables	PopTrans (***N =*** 127)				DIVAS (***N =*** 166)			
	n	%	%^**a**^	95%CI^**a**^	n	%	%^**a**^	95%CI^**a**^
**Serology**								
**HIV test**								
Negative	112	88.2	91.0	81.8-95-8	117	79	75.7	65.1-83.8
Positive	15	11.8	9.0	4.2-18.2	31	21	24.3	16.2-34.9
**Syphilis test**								
Negative	79	62.2	68.4	54.2-79.9	93	62	65.7	55.1-74.9
Positive	48	37.8	31.6	20.2-45.8	57	38	34.3	26.1-44.9
**Sociodemographic**								
**Age**								
< 25 years old	60	47.2	57.2	42.1-71.0	69	41.6	41.3	48.5-68.3
≥ 25 years old	67	52.8	42.8	29.0-57.9	97	58.4	58.7	48.5-51.6
**Years of schooling**								
≤ 9 years	49	38.6	26.7	16.6-40.1	64	38.8	38.6	29.0-49.1
> 9 years	78	61.4	73.3	59.9-83.4	101	61.2	61.4	50.9-71.0
**Skin color**								
White	22	17.3	18.9	9.1-35.1	21	13.3	11.0	6.1-19.0
Non-White	105	82.7	81.1	65.0-90.9	137	86.7	89.0	81.0-93.9
**Income (Brazilian minimum wage)**								
≤ $190.50	45	35.4	39.1	25.2-54.9	51	39.2	42.9	31.5-55.1
> $190.50	82	64.6	60.9	45.1-74.8	79	60.8	57.1	44.9-68.5
**Occupation**								
Formal and informal employment/self-employment	39	30.7	28.7	17.2-43.8	58	34.9	37.8	28.1-48.6
Sex work/unemployed	88	69.3	71.3	56.2-82.8	108	65.1	62.2	51.5-71.9
**Discrimination**								
**Gender based discrimination**								
No	26	20.5	16.6	9.5-26.5	21	12.6	15.1	8.4-25.5
Yes	101	79.5	83.7	73.5-90.5	145	87.4	84.9	74.5-91.6
**Forced sex**								
No	82	64.6	62.4	46.4-76.1	89	53.9	55.0	44.6-65.0
Yes	45	35.4	37.6	23.9-53.6	76	46.1	45.0	35.0-55.4
**Discrimination in health services**								
No	84	66.1	58.8	43.1-72.9	116	69.9	77.4	68.8-84.2
Yes	43	33.9	41.2	27.1-56.9	50	30.1	22.6	15.8-31.2
**Discrimination in the family**								
No	64	50.4	51.1	36.4-65.7	94	56.6	57.5	47.1-67.2
Yes	63	49.6	48.9	34.4-63.6	72	43.4	42.5	32.8-52.9
**Discrimination by neighbors**								
No	56	44.1	35.0	23.2-49.0	113	68.1	71.5	61.9-79.4
Yes	71	55.9	65.0	51.0-76.8	53	31.9	28.5	20.6-38.1
**Sexual behavior**								
**Condom use during receptive anal sex in the last 6 months**								
Always use	39	30.7	23.6	14.0-37.0	–	–	–	
Inconsistent use	88	69.3	73.4	63.0-86.0	–	–	–	
**Condom use during receptive anal sex in the last 30 days**								
Always use	–	–	–		38	28.6	25.2	16.6-36.3
Inconsistent use	–	–	–		95	71.4	74.8	63.7-83.4

^**a**^Weighted by RDS-II estimator

While 57.2% were younger than 25 years of age in the PopTrans study (2016), 58.7% were 25 years of age or older in the DIVAS study (2017). In both studies, most participants self-identified as non-white (81.1 and 89.0%), reported more than 9 years of schooling (73.3 and 61.4%), income greater than US$190.50 (60.9 and 57.1%) and were either engaged in sex work or unemployed (71.3 and 62.2%), respectively (Table 1).

Most reported facing gender-based discrimination (83.7 and 84.9%), nearly half reported discrimination in health services (41.2 and 22.6%), or discrimination in the family (48.9 and 42.5%). In the case of discrimination by neighbors, there were pronounced differences between the two studies: in the PopTrans study, most reported that they had discriminated by neighbors (65.0%), however in the DIVAS study this frequency was 28.5%. Regarding sexual behavior, nearly half reported they have been victims of sexual abuse (37.6 and 45.0%) and most reported inconsistency in condom use during receptive anal sex in the last 6 months (73.4%) and in the last 30 days (74.8%), respectively for the PopTrans and DIVAS studies (Table 1).

In the PopTrans study, the predictor variables associated with HIV infection (*p*-value< 0.050) were: income, occupation, gender-based discrimination as well as discrimination by the family and by neighbors. In DIVAS, the associated variables (*p*-value< 0.050) were: only discrimination and lifetime history of syphilis infection (Table 2).

**Table 2:** Factors associated with HIV infection among TGW in Salvador, Northeast Brazil

Variables	PopTrans (***N =*** 127)				DIVAS (***N =*** 166)			
	% ^**a**^	***p*** value ^**a**^	OR ^**a**^	95%CI ^**a**^	% ^**a**^	***p*** value ^**a**^	OR ^**a**^	95%CI ^**a**^
**Sociodemographic**								
**Age**		0.143				0.252		
< 25 years old	4.92		1.00	–	29.23		1.00	–
≥ 25 years old	12.10		2.66	0.68-10.34	18.10		0.53	0.18-1.58
**Years of schooling**		0.407				0.372		
≤ 9 years	7.40		1.00	–	29.55		1.00	–
> 9 years	13.47		1.94	0.39-9.74	20.83		0.63	0.22-1.76
**Skin color**		0.070				0.160		
White	2.30		1.00	–	42.03		1.00	–
Non White	10.59		5.03	0.73-34.77	20.96		0.37	0.09-1.56
**Income (Brazilian minimum wage)**		0.001				0.494		
≤ $190.50	1.49		1.00	–	13.84		1.00	–
> $190.50	13.84		25.64	2.14-52-72	19.83		1.54	0.44-5.90
**Occupation**		0.011				0.491		
Formal and informal employment/self-employment	2.11		1.00	–	19.86		1.00	–
Sex professional/unemployed	11.8		6.21	1.26-30.52	26.97		1.49	0.47-4.68
**Discrimination**								
**Gender based discrimination**		0.037				0.003		
No	1.53		1.00	–	4.11		1.00	–
Yes	10.48		7.54	0.81-69.57	28.19		9.17	1.68-50.14
**Forced sex**		0.607				0.065		
No	10.31		1.00	–	16.37		1.00	–
Yes	6.88		0.64	0.11-3.55	34.13		2.65	0.92-7.58
**Discrimination in health services**		0.239				0.911		
No	5.88		1.00	–	24.61		1.00	–
Yes	13.50		2.50	0.52-12.01	23.41		0.94	0.29-2.99
**Discrimination in family**		0.004				0.958		
No	2.45		1.00	–	24.11		1.00	–
Yes	15.89		7.52	1.57-36.02	24.62		1.03	0.37-2.89
**Discrimination by neighbors**		0.001				0.165		
No	1.39		1.00	–	28.14		1.00	–
Yes	13.12		10.71	1.91-59.96	15.30		0.46	0.15-1.40
**Sexual behavior**								
**Condom use during receptive anal sex in the last 6 months**		0.539						
Always use	12.54		1.00	–				
Inconsistent use	7.93		0.60	0.12-3.13				
**Condom use during receptive anal sex in the last 30 days**						0.259		
Always use					34.24		1.00	–
Inconsistent use					20.16		0.48	0.14-1.74
**Serology**								
**Syphilis test**		0.051				< 0.050		
Negative	4.67		1.00	–	17.67		1.00	–
Positive	18.46		4.63	0.89-24.12	37.43		2.79	0.99-7.91

^**a**^Weighted by RDS-II estimator

In the multivariate analysis, the variables that significantly increased the chance of HIV infection in the PopTrans study were: experiencing discrimination by the family (OR 8.22; 95%CI: 1.49-45.48), or by neighbors (OR 6.55; 95%CI:1.12-38.14), and having a lifetime history of syphilis infection (OR 6.56; 95%CI:1.11-38.65). In the DIVAS study, the predictor variables that significantly increased the chance of getting HIV infection were: experiencing gender-based discrimination (OR: 8.65; 95%CI:1.45-51.59), and having a lifetime history of syphilis infection (OR: 3.13; 95%CI:3.13-51.59) (Table 3).

**Table 3:** Factors associated with HIV infection among TGW in Salvador, Northeast Brazil

Variables	PopTrans		DIVAS	
	aOR ^**a**^	95%CI ^**a**^	aOR ^**a**^	95%CI ^**a**^
**Gender-based discrimination**				
No			1.00	–
Yes			8.65	1.45-51.59
**Forced sex**				
No			1.00	–
Yes			2.16	0.73-6.40
**Discrimination in family**				
No	1.00	–		
Yes	8.22	1.49-45.48		
**Discrimination by neighbors**				
No	1.00	–		
Yes	6.55	1.12-38.14		
**Syphilis test**				
Negative	1.00	–	1.00	–
Positive	6.56	1.11-38.65	3.13	1.45-51.59
**Hosmer-Lemeshow (p value)**	1.000		0.994	

^**a**^Weighted by RDS-II estimator

## Discussion

The study estimated disproportionately high rates of HIV and syphilis among TGW when compared to the Brazilian population in general, and underscore the role of social determinants for HIV infection, such as income and discrimination perpetrated by people in close contact (family and neighbors). We also observed the association between syphilis and HIV, which is a finding commonly already related in the literature.

### HIV Prevalence

This study indicated a high prevalence of HIV in PopTrans (9.0%), and even higher in DIVAS (24.3%) among TGW in the city of Salvador, when compared to the general female population of Brazil (0.4%) [10]. Other studies with TGW conducted in Latin America show even higher prevalence rates. In Argentina, a survey identified that 34.1% (95%CI:28.7-39.9) of TGW were infected with HIV [41]. In Uruguay, Russi et al. [42] reported an HIV prevalence as high (21.5%) among TGW. Corroborating the aforementioned estimates, a meta-analytic study estimated a pooled HIV prevalence in Argentina and Uruguay of 33.5 and 18.8%, respectively [3].

Of note, these estimates from PopTrans and DIVAS are lower than estimates from studies using serological testing in some Brazilian cities, but similar to estimates from studies conducted in the same region. For example, a study carried out with 284 TGW from southern Brazil (2015) recruited from a health service in Porto Alegre estimated a prevalence of HIV infection of 25% [6]. In Rio de Janeiro (2017), located in southeast Brazil, a point prevalence of 31.2% (CI95% 18·8–43·6) was estimated in an RDS study of 345 TGW [7]. In contrast, studies from the same region where PopTrans and DIVAS were conducted found similar rates of HIV, albeit based on self-reported outcomes. For example, a 2008-2009 study carried out through the RDS recruitment of 110 *travestis* in the capital city of Recife and its metropolitan region identified a self-reported prevalence of 12.7% [8]; whereas another survey carried out in the capital city of Fortaleza in 2008, based on an RDS sample of 304 *travestis*, observed a self-reported prevalence of 12% [9]. The prevalence of these two cities in the Northeast is similar to that of the study in Salvador.

The prevalence differences found in both the PopTrans and DIVAS studies have no a simple explanation. Both studies were performed in same city, with same methods, and both studies may have used similar samples, but we cannot guarantee that they are exactly the same individuals. However, two hypotheses should be considered: the natural increase in the prevalence of infectious diseases that have no cure [43], and a non-probabilistic sample which may bias an external validity [44]. Significant progress has yet to be fully achieved as methods incorporating transportability have been progressively refined and applied to different populations in recent publications [45]. The same reasoning applies to our studies whereby small sample sizes are combined with larger geographical areas than Salvador, Bahia.

### Gender Based Discrimination

This study shows that gender-based discrimination increased the chance of HIV infection. This form of discrimination can be defined by moral, physical or psychological violence faced by TGW as a consequence of patriarchal and sexist societies [11, 46, 47]. Thus, discrimination is reported as a structural factor that exacerbates vulnerability of TGW to HIV, such as preventing access to health services promoting HIV prevention and care [11, 48, 49]. In the peer-reviewed literature, discrimination is also reported as a factor influencing vulnerability to HIV through association with risk behaviors, such as exposure to condomless anal sex which can hinder the negotiation of safe practices during receptive anal sex or access to condom [50–53].

The literature has pointed to sexual assault by family members [54, 55], as well as physical and sexual abuse, as factors associated with HIV infection among TGW [56]. Studies have also reported different forms of violence perpetrated by neighbors [55] and family [54, 55]. These experiences of abuse and discrimination has been shown to impact TGW across their life cousre and ofen starts within the family through rejection [19, 55, 57–69] and expulsion from home [60, 62, 64, 67] causing some TGW to be unstably housed [64, 66] and cause intense geographical displacement [70] and entry into sex work [15, 19, 50, 58, 61, 62, 64, 67, 70–72], a risk factor also highlighted in our study.

### Syphilis

Having had syphilis in life also significantly increased the chance of HIV infection among TGW in both studies. Syphilis is a commonly HIV-related infection because it increases the transmissibility of the infection and viral load in infected individuals [22]. From a biological point of view, micro lesions generated by infectious processes act as a gateway to HIV and, thus, increase the risk of transmission. Furthermore, both pathogens have similar transmission mechanisms hence the increased chance to acquire both HIV and other STIs such as syphilis [22, 73, 74]. Further, syphilis can also be viewed as a marker to HIV vulnerability: both syphilis and HIV infection have similar risk factors such as condomless sex or inconsistent condom use, sex work, and other sexual risk behaviors [75, 76]. Moreover untreated syphilis increases the risk of HIV infection significantly as the lesions as a gateway to infection and raises the risk of transmission by the increasing viral load in an already co-infected partner [75, 77, 78].

### Limitations and Strengths

This study has some limitations. Our study (like all other RDS studies) uses non-probability samples and invariably recruit a small number of participants. Non-probability samples are always associated with challenges to statistical inference [79]. Small samples are never free of potential beta type errors or, as recently proposed, by Rothman and Greenland [80] lack the so desired precision (as made evident by large confidence intervals). On the other hand, local studies are an invaluable source of information for pooled analyses. The latter have been found to be accurate and a key asset for public policies as mentioned.

Further, the cross-sectional design does not allow us to define a clear temporal relationship between what we have defined as predictor variables and the outcome. With some exceptions for associations presenting a logical sequence over time, reverse causality is sometimes difficult to discern. The sampling methodology used, RDS, prevents the data from this study from being generalized, despite the promises offered by both innovative methods on re-weighting non-probability samples (for instance, the use of pseudo weights; see the previously mentioned paper by Elliott and Valliant (2017), and the brand-new perspectives emerging from developments in the field of transportability. The latter has been found to be robust respecting the generalizability of randomized clinical trials [81]. Still, the contextual dimensions, especially when highly stigmatized populations have been targeted in specific contexts, remains a major challenge for transportability. Although generalizability is still a goal, it should be considered as a cost of ignoring the long tradition of understanding complex contexts or in the words of Zinberg [82], to take into consideration individuals, sets, and settings.

Finally, selection bias may be an issue, as the seeds invited their peers in a chain [34]. However, the adjustment used by RDS reduces this bias and makes it possible to generate important information about the network. As is common with populations that are difficult to access, several other studies have used the RDS design for these populations as the most feasible option [7, 12, 16, 83–85].

## Conclusion

The prevalence of HIV infection among TGW remains high. Even as Brazilian policies on HIV/AIDS and other STI have progressed over the years, these data indicate the urgency of intersectoral actions on the determinants of vulnerability to HIV/AIDS. Further it underscores the need for health interventions to develop TGW-specific prevention, treatment and other health services or programs, especially with regard to addressing gender-based discrimination in general, and specifically among family and neighbors. Gender-based discrimination has been increasingly reported as a key factor severely limiting access to existing health services and programs among TGW and other gender discordant population. Our study reinforces the need to implement policies that combat discrimination in general and specifically in the context of health services and programs. Only in so doing can we curb new infections and simultaneously address the broader context of social vulnerability that permeates the daily experience of TGW in Brazil and beyond.

## Supplementary Information

**Additional file 1.**

**Additional file 2.**

## Data Availability

The datasets analyzed during the current study are available in the Havard Dataverse repository, 10.7910/DVN/PD7D5U.
