# Supplementary material for: HIV prevalence among transgender women in Northeast Brazil – Findings from two Respondent Driven Sampling studies
Source: BMC Public Health. 2022 Nov 18;22:2120. doi: 10.1186/s12889-022-14589-5 (PMC9673344; doi:10.1186/s12889-022-14589-5)
Supplement: Supplementary file 1 — Additional file 1. [file 12889_2022_14589_MOESM1_ESM.docx]

**Correlation matrix and Variance Inflation Factor (VIF).**

| **PopTrans** | | | | | | |
| --- | --- | --- | --- | --- | --- | --- |
| **Variables** | **I** | **II** | **III** | **IV** | **V** | **VIF** |
| **Gender-based discrimination (I)** | 1.00 |  |  |  |  | 1.38 |
| **Forced sex (II)** | 0.21 | 1.00 |  |  |  | 1.09 |
| **Discrimination in family (III)** | 0.35 | 0.15 | 1.00 |  |  | 1.24 |
| **Discrimination by neighbors (IV)** | 0.49 | 0.26 | 0.41 | 1.00 |  | 1.48 |
| **Syphilis test (V)** | 0.03 | 0.07 | 0.01 | 0.01 | 1.00 | 1.01 |
| **DIVAS** | | | | | | |
| **Variables** | **I** | **II** | **III** | **IV** | **V** | **VIF** |
| **Gender-based discrimination (I)** | 1.00 |  |  |  |  | 1.21 |
| **Forced sex (II)** | 0.21 | 1.00 |  |  |  | 1.06 |
| **Discrimination in family (III)** | 0.33 | 0.17 | 1.00 |  |  | 1.15 |
| **Discrimination by neighbors (IV)** | 0.26 | 0.17 | 0.21 | 1.00 |  | 1.10 |
| **Syphilis test (V)** | -0.07 | 0.01 | -0.07 | -0.11 | 1;00 | 1.02 |
