# Supplementary figures and images for: HIV prevalence among transgender women in Northeast Brazil – Findings from two Respondent Driven Sampling studies
Source: BMC Public Health. 2022 Nov 18;22:2120. doi: 10.1186/s12889-022-14589-5 (PMC9673344; doi:10.1186/s12889-022-14589-5)

**Venn diagram for discrimination variables**


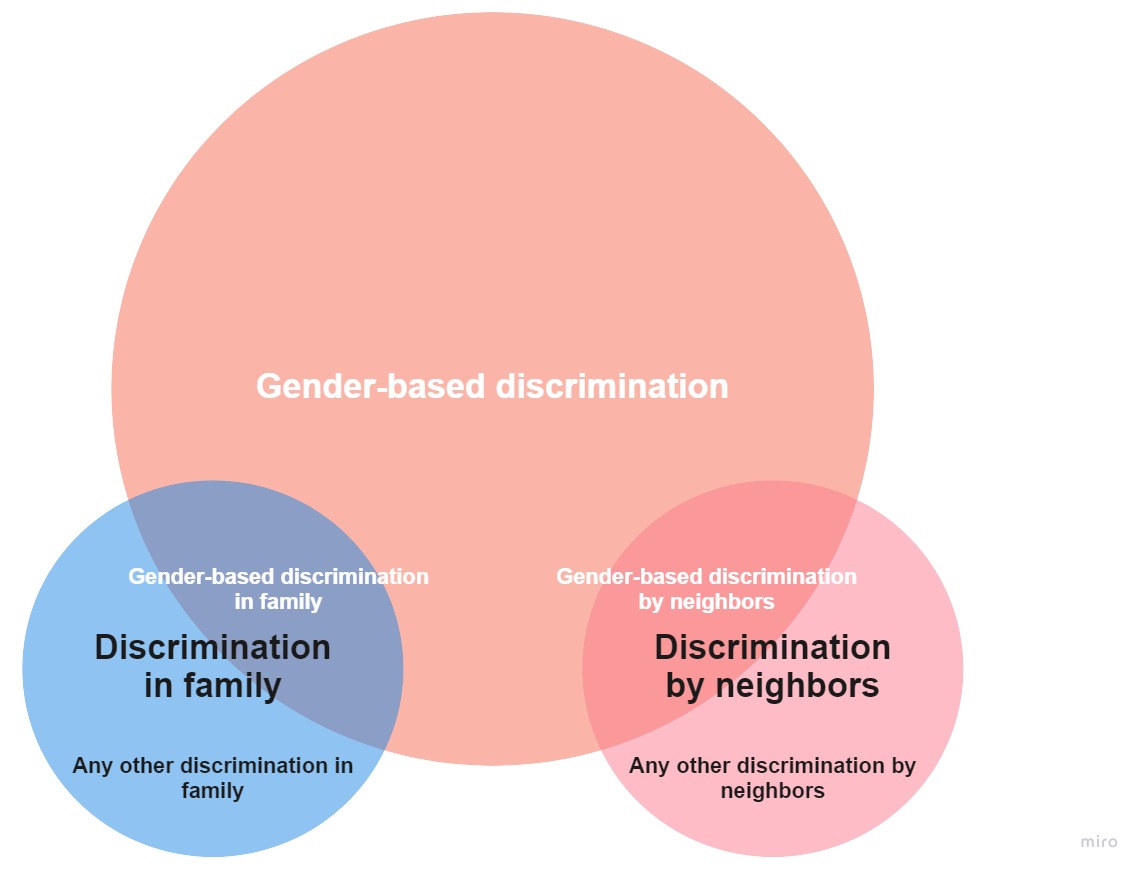

Supplement: Supplementary file 2 — Additional file 2. [file 12889_2022_14589_MOESM2_ESM.docx]
